# Emotion norms for 6000 Polish word meanings with a direct mapping to the Polish wordnet

**DOI:** 10.3758/s13428-021-01697-0

**Published:** 2021-12-10

**Authors:** Małgorzata Wierzba, Monika Riegel, Jan Kocoń, Piotr Miłkowski, Arkadiusz Janz, Katarzyna Klessa, Konrad Juszczyk, Barbara Konat, Damian Grimling, Maciej Piasecki, Artur Marchewka

**Affiliations:** 1 Laboratory of Brain Imaging, Nencki Institute of Experimental Biology, Polish Academy of Sciences, 3 Pasteur Street, 02-093 Warsaw, Poland; 2 Department of Psychology, Columbia University, New York, NY USA; 3 Department of Artificial Intelligence, Wrocław University of Science and Technology, Wrocław, Poland; 4 Faculty of Modern Languages and Literatures, Adam Mickiewicz University, Poznań, Poland; 5 Faculty of Psychology and Cognitive Sciences, Adam Mickiewicz University, Poznań, Poland; 6 Sentimenti Sp. z o.o., Poznań, Poland

**Keywords:** Emotion, Valence, Arousal, Emotion categories, Sentiment analysis, Words, Word meanings, Word senses, Wordnet

## Abstract

Emotion lexicons are useful in research across various disciplines, but the availability of such resources remains limited for most languages. While existing emotion lexicons typically comprise words, it is a particular meaning of a word (rather than the word itself) that conveys emotion. To mitigate this issue, we present the Emotion Meanings dataset, a novel dataset of 6000 Polish word meanings. The word meanings are derived from the Polish wordnet (plWordNet), a large semantic network interlinking words by means of lexical and conceptual relations. The word meanings were manually rated for valence and arousal, along with a variety of basic emotion categories (anger, disgust, fear, sadness, anticipation, happiness, surprise, and trust). The annotations were found to be highly reliable, as demonstrated by the similarity between data collected in two independent samples: *unsupervised* (*n* = 21,317) and *supervised* (*n* = 561). Although we found the annotations to be relatively stable for female, male, younger, and older participants, we share both summary data and individual data to enable emotion research on different demographically specific subgroups. The word meanings are further accompanied by the relevant metadata, derived from open-source linguistic resources. Direct mapping to Princeton WordNet makes the dataset suitable for research on multiple languages. Altogether, this dataset provides a versatile resource that can be employed for emotion research in psychology, cognitive science, psycholinguistics, computational linguistics, and natural language processing.

**Supplementary Information:**

The online version contains supplementary material available at 10.3758/s13428-021-01697-0.

## Introduction

As humans we have the remarkable capacity to express complex, nuanced emotions with language. While this notion has inspired researchers across multiple domains, scientific investigation of this topic remains highly challenging. A standard procedure for studying emotions expressed in natural language is to use existing lexicons or sets of words, whose emotional properties are already known. These lexicons typically comprise words characterized in terms of emotion attributes derived from one of the dominant theoretical frameworks: *dimensional* or *categorical*. According to the former, each emotional state can be represented by its location in a multidimensional space, where valence or polarity (ranging from *negative* to *positive*) and arousal (ranging from *low* to *high*) explain most of the observed variance (Bradley & Lang, 1994; Osgood et al., 1957). A competing account distinguishes several categories, referred to as *basic emotions*, such as *anger*, *disgust*, *fear*, *sadness*, *anticipation*, *happiness*, *surprise*, and *trust*(Ekman, 1992; Ortony & Turner, 1990; Plutchik, 1982). These categories represent elementary states, some combination of which gives rise to more complex emotions. Since there have been various interpretations of the concept of basic emotions, different theories stipulate different numbers of such elementary states, with Ekman’s model (Ekman, 1992) and Plutchik’s model (Plutchik, 1982) being most popular. Both theoretical accounts (dimensional and categorical) have gained comparable recognition in the scientific community. Therefore, datasets of words characterized in line with both accounts are in high demand, as they make it possible to extend the scope of research questions that can be addressed.

We can identify two major lines of research that can benefit from the use of emotion lexicons. The first one concerns the psychology of emotion, as well as its role in other cognitive processes. Here, information obtained from the existing emotion lexicons can be used either to directly study the impact of emotion on the processing of words, or to control for possible confounding effects of emotion on other processes. In such cases, a limited number of stimuli that vary with respect to one factor and are matched on other factors are usually sufficient. On the other hand, it is important to use stimuli whose emotional features can be reliably estimated. Datasets used for this kind of research are typically created by asking people to manually rate words, one by one, with respect to several properties. Obviously, the procedure of obtaining such ratings for a large number of words can be very expensive and time-consuming, as multiple persons have to rate each word in order to reliably estimate the emotional meaning conveyed by it. As a result, most available emotion lexicons are relatively small. For instance, the Affective Norms for English Words (ANEW; Bradley & Lang, 2017), likely one of the most commonly used datasets of emotion ratings in English, contains merely 1034 words. A more recent dataset provides ratings for 13,915 English words (Warriner et al., 2013). Unlike datasets of non-verbal stimuli (e.g. images, videos), which can be used to study populations drawn from different cultures, datasets of verbal stimuli (e.g. words, sentences, paragraphs) are culturally specific. Therefore, research involving verbal stimuli is limited by the availability of suitable resources in different languages. Such resources have already been created for, among others, Dutch (4300 words; Moors et al., 2013), Finish (420 words; Eilola & Havelka, 2010), French (1031 words; Monnier & Syssau, 2014), German (2900 words; Briesemeister et al., 2011; Võ et al., 2006, 2009), Italian (1034 words; Montefinese et al., 2014), Polish (4900 words; Imbir, 2015, 2016; 2902 words; Riegel et al., 2015; Wierzba et al., 2015), Portuguese (1034 words; Soares et al., 2012), and Spanish (1034 words; Redondo et al., 2007). Yet, emotion ratings for these languages are relatively scarce. Moreover, most of these datasets were created by translating other such resources (typically the 1034 words included in the original ANEW dataset). Hence, the rules governing the selection of words make these datasets hardly representative of the entire lexicon of any given language. Moreover, in most of these datasets no distinctions are made between various meanings of individual words, as if the word itself, rather than its particular meaning, conveyed a certain emotion.

Another, somewhat different, line of research focuses on the endeavor to automatically detect emotion in natural language. In this sort of research, emotion lexicons are used to inform computational models that process large amounts of text, such as tweets (Cody et al., 2015; Gallagher et al., 2018; Kiritchenko et al., 2014), newspaper articles (Reagan et al., 2017), or books (Reagan et al., 2016). Here, the richer the prior knowledge about each word, the more reliable the model for the estimation or prediction of the emotional value of a specific text. Thus, datasets used for developing such models typically comprise many more words and—by means of rich, extensive information on a variety of linguistic features, such as semantic relations between different words and their meanings—support the approximation of the emotional value of each word belonging to the same language. Such datasets typically cover thousands of words, lemmas, lexemes, or other elementary units of language (e.g. Dodds et al., 2015; Mohammad, 2016), which are sometimes directly linked to large databases of naturally occurring language, called corpora. Due to the number of words comprising such datasets, the ratings are usually given by a small number of trained annotators (e.g. 2–3 people) that typically have expert knowledge in linguistics or natural language processing. It is also quite common to use such manually rated words to automatically estimate emotion values for other words based on semantic similarity or associations between words (Van Rensbergen et al., 2016). Such an approach allows one to obtain emotion values for datasets significantly larger than those created through manual rating. Despite the growing ease with which data can be collected, large emotion datasets are only available in a relatively small number of languages, mostly in English (but see Dodds et al., 2015 for exceptions). Most of these datasets are limited to characterizations of words in terms of emotion in a rather broad sense (typically in terms of polarity, as either negative or positive), disregarding the complexity of emotions that can be expressed with language (but see Mohammad & Turney, 2013; Mohammad & Turney, 2010 for exceptions). While the availability of such large-scale resources can certainly advance research across multiple domains, much depends on the quality of the data. Thus, validating automatically derived resources against human-generated data seems crucial (Brysbaert et al., 2017).

In the present work we combine the insights contributed by various disciplines of research to introduce the Emotion Meanings dataset, a novel resource containing 6000 Polish word meanings annotated in terms of emotion. The word meanings were carefully selected from an initial pool of over 30,000 word meanings, so as to best represent distinct basic emotions: anger, disgust, fear, sadness, anticipation, happiness, surprise, and trust (Ekman, 1992; Plutchik, 1982). Drawing on psychology and cognitive science, the word meanings included in the present dataset were manually rated to provide reliable measures of valence and arousal, along with a variety of basic emotion categories. The ratings come from a large, demographically diverse group of 21,317 participants who completed the task online, but were also validated on an independent group of 561 participants who came to the laboratory in person. This step was crucial to ensure the high quality of the data. Importantly, the ratings are available both as summary scores and as individual scores to enable research on demographically specific subgroups, differing in terms of gender, age, education, and other factors. Drawing on corpus linguistics and natural language processing research, the present dataset comprises words directly linked to the precise indications of meaning, derived from the Polish wordnet (plWordNet, Słowosieć^1^). plWordNet is a large and comprehensive relational dictionary, which reflects the lexical system of the Polish language and currently contains 285,000 word meanings, linked with each other by over 600,000 semantic relations (Dziob et al., 2019; Piasecki et al., 2009). By having its roots in plWordNet, the present dataset can also be easily mapped to other languages, as long as the mapping between respective wordnets is available. For a start, we supplement the present dataset with its mapping to the Princeton WordNet^2^ for English. Finally, all word meanings in the dataset are accompanied by the relevant metadata derived from other open-source resources. As such, the present dataset is a unique resource that hopefully addresses some of the limitations of previous such datasets available in the Polish language (Imbir, 2015, 2016; Riegel et al., 2015; Wierzba et al., 2015). The dataset is publicly available for scientific, non-commercial use with the aim of stimulating further research across many disciplines, including psychology, cognitive science, psycholinguistics, computational linguistics, or natural language processing.

## Methods

The dataset described in this article was collected within a large research and development project, in which 30,080 word meanings were annotated in terms of emotion. The data for 6000 word meanings, described here, are shared with the broad research community for non-commercial use. The remaining data are subject to copyright restrictions.

### Materials

The initial pool of 30,080 word meanings was selected from the Polish wordnet (plWordNet), a large relational semantic dictionary which reflects the lexical system of the contemporary Polish language. The plWordNet currently contains 285,000 word meanings, linked with each other by over 600,000 semantic relations.

Essentially, each word in the plWordNet is directly linked to its meaning. Moreover, thanks to the mapping between plWordNet and Princeton WordNet, each word is further linked to its English equivalent. The details of the mapping procedure were presented in Rudnicka et al. (2021). The mapping was created manually by experienced bilingual lexicographers working under the supervision of senior lexicographers. The WordNetLoom project (Naskręt et al., 2018) was used as a main platform for supporting the lexicographers during their work. The procedure consisted of three steps: (1) recognition of the source word meaning in one language, (2) searching target word meaning candidates in another language, and (3) selecting a target word meaning and a type of cross-lingual relation. The final mapping contains almost 300,000 cross-lingual relations^3^ between Polish and English word meanings. The resource is available under an open wordnet license^4^ and it is widely used in many tools and language resources such as CloudNet Word Cloud Generator, Google Translate, BabelNet (Navigli & Ponzetto, 2012), Ling.pl^5^, or Open Multilingual Wordnet (Bond & Foster, 2013), see Rudnicka et al. (2021) for more details.

Each word meaning in plWordNet has a unique identifier, and is represented by the pair of (1) a lemma (a canonical form of a word), such as *wirus* (“virus”), and (2) a sense (a particular meaning in which the word is used), such as *wirus-1* (denoting “an ultramicroscopic infectious agent that replicates itself only within cells of living hosts”)^6^ or *wirus-2* (denoting “a software program capable of reproducing itself and usually capable of causing great harm to files or other programs on the same computer”)^7^. Furthermore, each entry is mapped to its English equivalent, in this case *virus-1*^8^ or *virus-3*^9^, respectively.

For the purpose of the present project, we selected 30,080 word meanings from plWordNet. The selection was based on the results of the plWordNet-emo project (Janz et al., 2017), where more than 87,000 word meanings were annotated with valence, emotions, as well as fundamental values (Kocoń et al., 2019), covering 54,000 synsets (i.e. sets of word meanings representing the same concept). We used the following criteria for the selection process (Kocoń et al., 2019): (1) we chose non-neutral word meanings first; (2) the maximum number of selected word meanings belonging to one synset was 3; (3) the degree of the synset node containing a word meaning (number of relations to other synsets) in the plWordNet graph was in the range of 3–6.

Finally, for each word meaning, a short phrase was manually created by a group of experienced wordnet editors. The purpose of these phrases was to direct future study participants to specific meanings of rated words. For example, the phrases *computer virus* and *deadly virus* can be used to distinguish between different meanings of the same word *virus.*

### Participants

Two separate studies were conducted: *unsupervised* and *supervised*. In the former case, participants (*n* = 21,317) completed the task remotely and worked without any supervision. In the latter case, participants (*n* = 561) came to the laboratory in person, where their work was monitored and where they were offered assistance as needed. Volunteers were recruited through a mass mailing, targeting a wide group of respondents. Participants in the *unsupervised* group received compensation in the form of virtual currency that could be exchanged for small gifts. Participants in the *supervised* group received financial reward of roughly equivalent value. Only native Polish speakers were permitted to join the study. Furthermore, stratified sampling was used in order to reach a demographically diverse group of participants. Specifically, the stratification was defined based on gender (male, female) and age (18–34 years old, 35–64 years old) to reflect the demographic profile of the population of Poland^10^, based on data available at the time of the study. Additionally, we collected other demographic data, including place of residence, education, relationship status, employment status, income, as well as political views. The full demographic questionnaire (the original Polish version, as well as the English translation) can be found in the supplementary materials. Although the stratification criteria used in both studies were the same, it is worth noting that the two samples differed significantly in terms of their demographic characteristics, as assessed by means of the Pearson’s chi-square test (*p* < 0.001 for all reported variables). However, in each case, the effect size was rather small, as measured by Cramer’s V (*φ*_*c*_ < 0.1 for all reported variables). In other words, both samples had fairly similar demographic characteristics. Importantly, though, the *unsupervised* group was much more heterogeneous than the *supervised* group in terms of place of residence. Further information about the study samples can be found in Table 1.

**Table 1:** Demographic profile of the samples recruited for the *unsupervised* (*n* = 21,317) and the *supervised* (*n* = 561) study

Demographic characteristics		*Unsupervised* (%)	*Supervised* (%)	Statistics		
				*χ*^*2*^	*p*	*φ*_*c*_
Gender	Male	40.34	49.73	20.0	< 0.001*	0.0302
	Female	59.66	50.27			
Age (years)	18–24	12.91	13.46	53.3	< 0.001*	0.0498
	25–34	30.94	23.88			
	35–44	26.06	20.29			
	45–54	17.25	20.47			
	55–64	12.84	21.90			
Place of residence	Pop. ≤ 20,000	24.16	–	–	–	–
	Pop. 20,001–50,000	20.58	–			
	Pop. 50,001–100,000	12.70	–			
	Pop. 100,001–200,000	11.11	–			
	Pop. 200,001–500,000	12.93	–			
	Pop. > 500,000	18.52	100			
Education	No formal education	0.17	0.37	52.8	< 0.001*	0.0539
	Incomplete primary	0.18	0.37			
	Primary	1.11	1.47			
	Lower secondary	1.33	2.58			
	Basic vocational	8.68	7.55			
	Incomplete secondary	3.36	4.60			
	Secondary	26.26	32.97			
	Post-secondary	10.12	6.26			
	Undergraduate	8.13	5.16			
	Incomplete higher	3.21	5.34			
	Higher	36.24	31.86			
	Doctoral degree	1.11	0.92			
	Other	0.10	0.55			

### Procedure

The data were obtained in the course of two independent studies: *unsupervised* and *supervised*. In the *unsupervised* study, each of the 30,080 word meanings was rated by 55.76 people on average (ranging between 47 and 138). In the *supervised* study, some of those word meanings (2997 out of 30,080 word meanings) were rated by another 26.08 people on average (ranging between 23 and 28) each. Individual participants were allowed to complete up to three rating sessions, each comprising 50 word meanings. The word meanings to be rated in a given rating session were randomly selected from the initial pool of word meanings. However, word meanings with the smallest number of ratings collected so far had greater chance of being selected.

The procedures used in the two studies (*unsupervised* and *supervised*) were as closely matched as possible. In the following sections, we provide details of both studies for transparency.

### Data collection in the *unsupervised* study

Participants worked remotely at a place of their choice. They received detailed task instructions, but worked without any further supervision. In case of any technical issues, they were able to ask for assistance using a dedicated email address. Participants were invited to complete up to three consecutive rating sessions. Invitation to the next session was issued no earlier than 24 hours after the previous session had been completed. The financial reward was increased with every session to motivate participants to complete the study.

### Data collection in the *supervised* study

Participants were invited to a research laboratory equipped with computer rooms specifically designed to reduce and control distraction. Once the identity of each person was verified, they were assigned a unique identifier. Individuals worked in small groups of up to 12 people, each person working individually on a separate computer station. A research assistant was present in the room to assist participants in solving any technical problems and to monitor their work. Participants were required to complete three consecutive rating sessions, with an obligatory break in between. They received compensation after completing all three sessions.

### Details of the rating task

A purpose-built, secure web application was used to collect the ratings. The task instructions in both studies were identical. The participants were aware of the general purpose of the study. They were informed that they would be asked to rate 50 words and that their responses would be used to create a large emotion lexicon for Polish. Furthermore, participants were notified that each word would be rated on 10 scales.

The bounds of the scales were explicitly defined in the following way: valence (from −3: *the word is associated with strong negative emotions*, through 0: *the word is not associated with any emotions*, to 3: *the word is associated with strong positive emotions*); arousal (from 0: *the word is not associated with any emotions*, to 4: *the word is associated with strong arousal (e.g. excitement, restlessness)*); basic emotions: anger, disgust, fear, sadness, anticipation, happiness, surprise, and trust (from 0: *the word is not associated with this emotion*, to 4: *the word is strongly associated with this emotion*). During the assessment task, these explicit descriptions were no longer present on screen, for practical reasons. Instead, the scales were labeled with names and numeric values. Moreover, the bounds of some of the scales were further marked with short labels: valence (from −3: *negative*, to 3: *positive*), arousal (from 0: *low*, to 4: *high*). Importantly, it should be noted that we used different bounds for valence and arousal scales. While valence is best represented by a bipolar scale with negative values on one side of the scale and positive values on the other side of the scale, arousal is better understood as a unipolar scale with increasing positive values (Riegel et al., 2015; Võ et al., 2006, 2009).

During the assessment task, each trial began with a brief display of a word, together with a short phrase to indicate the intended meaning of the word. Next, on the following screen, the participants were still able to see the word and the phrase in the upper part of the screen, but this time, they were asked to rate the word in terms of valence, arousal, as well as basic emotions. As soon as all the responses were submitted, the next trial would begin. There was no time limit to complete the task, but the participants were encouraged to indicate their immediate reaction to the words. The participants were able to return to the instruction screen at any time during the session.

### Data preprocessing

In total, 16,771,960 individual ratings were contributed by 21,317 people in the *unsupervised* study, and 781,510 individual ratings were contributed by 561 people in the *supervised* study. First, we discarded data from sessions that were not completed due to technical issues or the participant’s decision. Only sessions with at least 50 word meanings rated were regarded as completed.

Next, the data from all the sessions were pooled. Each record corresponds to a unique combination of a plWordNet identifier and a participant identifier, to indicate both how each individual word was rated by each person, as well as to provide additional demographic information on the participant’s gender, age, place of residence, education, relationship status, employment status, income, as well as political views. Such organization allows for data to be reused to investigate demographically specific subgroups.

Finally, the data were aggregated to provide summary scores (means and standard deviations) of valence, arousal, anger, disgust, fear, sadness, anticipation, happiness, surprise, and trust ratings for each word. Thus, each record corresponds to a unique plWordNet identifier and provides means and standard deviations of each measure of interest, together with the number of people who contributed the data. The summary scores were calculated based on responses of all participants, as well as based on responses contributed by several demographically specific groups: female, male, younger, and older individuals.

Furthermore, we provide information on each word represented by a unique plWordNet identifier: the corresponding Polish word (lemma), the corresponding phrase, length (number of characters), and frequency of use of a given word (lemma). Finally, for each word, we provide a direct mapping to the Princeton WordNet.

The steps described above were performed separately for the data from the *unsupervised* study and for the data from the *supervised* study to facilitate further comparisons.

### Selection criteria

Our goal was to make sure that all the basic emotions are well represented in the Emotion Meanings dataset. Since each individual word was rated in terms of the intensity of each basic emotion (anger, disgust, fear, sadness, anticipation, happiness, surprise, and trust), it could be associated mostly with one dominant emotion (e.g., happiness), but could in principle be associated with several emotions (e.g., anger and disgust) or with none.

To select the word meanings, we adopted a method introduced in our previous work (Wierzba et al., 2015). Here, we consider an eight-dimensional hypercube, with each axis corresponding to one of the basic emotions. The ratings of a given word determine its position in the hypercube. Eight of the hypercube's corners represent the *emotion classes*: [4 0 0 0 0 0 0 0] anger, [0 4 0 0 0 0 0 0] disgust, [0 0 4 0 0 0 0 0] fear, [0 0 0 4 0 0 0 0] sadness, [0 0 0 0 4 0 0 0] anticipation, [0 0 0 0 0 4 0 0] happiness, [0 0 0 0 0 0 4 0] surprise, and [0 0 0 0 0 0 0 4] trust. The origin, namely [0 0 0 0 0 0 0 0], represents the *neutral class*. The distance of each word from each of the corners can be calculated using the standard formula:

$$d\left(p,q\right)=\sqrt{{\left(\ {p}_1-{q}_1\ \right)}^2+{\left(\ {p}_2-{q}_2\ \right)}^2+\dots +{\left(\ {p}_k-{q}_k\ \right)}^2}.$$

The distances are first calculated participant-wise based on the individual ratings contributed by each person. Next, the distances are averaged over all participants to yield a summary measure of distance of each word from each of the corners:

$$ \overline{d}=\frac{1}{n}\left({\sum}_{i=1}^n{d}_i\right)=\frac{d_1+{d}_1++{d}_n}{n}\cdotp $$

The following conditions must be fulfilled in order for a word to be assigned to one of the classes: (1) the word’s distance to the respective corner must be smaller than a certain threshold; (2) the word must meet the first condition for one class only; (3) if the word falls within an area of intersection of two (or more) classes, it remains unclassified; and (4) similarly, if the word does not meet the first condition for any of the classes, it remains unclassified.

As outlined above, this method can be used flexibly, depending on one's needs. One approach is to set a certain threshold value for each class. This would in turn determine the size of each class (the number of word meanings). Another approach is to set a certain class size (the number of word meanings) for each class. This would require a specific combination of threshold values to produce classes of the desired sizes.

Here, the initial pool of 30,080 word meanings was examined to select 6000 word meanings in total: 5000 word meanings for which one dominant emotion could be identified (eight *emotion classes*, of equal size), as well as 1000 neutral word meanings (*neutral class*). The threshold values were determined with the use of a simple genetic algorithm.

## Results

Here, we will use the following abbreviations to denote classes of word meanings: ANG, anger; DIS, disgust; FEA, fear; SAD, sadness; ANT, anticipation; HAP, happiness; SUR, surprise; TRU, trust; NEU, neutral. Otherwise, we will use full terms to denote measured variables: valence, arousal, anger, disgust, fear, sadness, anticipation, happiness, surprise, and trust.

### General description of the dataset

We examined the distribution of mean ratings for each measured variable. We used ratings obtained in the *unsupervised* study, as these data were available for all the 6000 word meanings included in the present dataset. The word meanings were rated by 50.52 people on average (*min* = 38, *max* = 61). The sample size for each class of word meanings separately is summarized in Table 2.

**Table 2:** Sample size for the word meanings as obtained in the *unsupervised* and the *supervised* study. Sample size is considered for each emotion class separately, as well as in total

Word meanings	Number of word meanings	*mean N*	*min N*	*max N*
ANG class	625	50.40	39	58
DIS class	625	50.49	41	61
FEA class	625	50.38	39	58
SAD class	625	50.32	40	61
ANT class	625	50.67	41	58
HAP class	625	50.61	41	60
SUR class	625	50.22	39	61
TRU class	625	50.42	38	59
NEU class	1000	50.96	41	60
Total *unsupervised*	6000	50.52	38	61
Total *supervised*	634	24.69	20	28

*ANG* anger, *DIS* disgust, *FEA* fear, *SAD* sadness, *ANT* anticipation, *HAP* happiness, *SUR* surprise, *TRU* trust, *NEU* neutral, *N* sample size, *min* minimum, *max* maximum

**Fig. 1:**
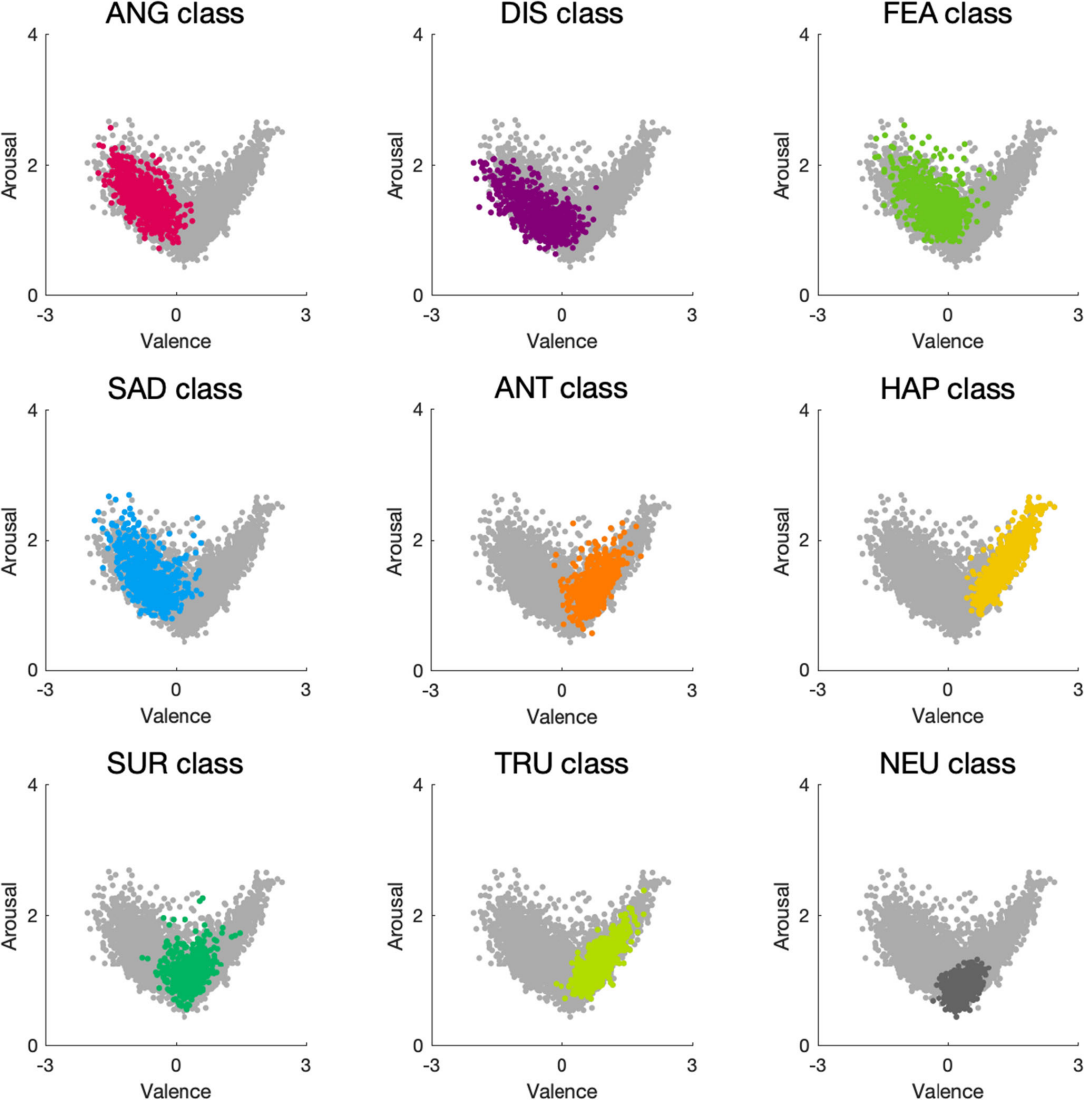
Distribution of the mean valence and arousal ratings for word meanings assigned to each class. In each case, the darker color represents word meanings belonging to a given class, whereas the light gray represents the remaining word meanings. Abbreviations: ANG, anger; DIS, disgust; FEA, fear; SAD, sadness; ANT, anticipation; HAP, happiness; SUR, surprise; TRU, trust; NEU, neutral

The summary of emotion ratings obtained for word meanings assigned to each class is provided in Table 3. The distribution of mean valence and arousal ratings for each class is depicted in Fig. 1, as well as in Supplementary Figures 1 and 2. Furthermore, the distribution of mean anger, disgust, fear, sadness, anticipation, happiness, surprise, and trust ratings for each class is provided in the Supplementary Figure 3. As demonstrated in Fig. 1, we observed a nonlinear relationship between valence and arousal: (1) word meanings that were rated more extreme in terms of valence (either more negative or more positive) were also rated as more arousing; (2) word meanings that were rated as neutral in terms of valence were also rated as less arousing. This finding is in agreement with many previous studies (Bradley & Lang, 2007, 2017; Eilola & Havelka, 2010; Imbir, 2015, 2016; Monnier & Syssau, 2014; Montefinese et al., 2014; Moors et al., 2013; Redondo et al., 2007; Riegel et al., 2015; Soares et al., 2012; Võ et al., 2006, 2009; Warriner et al., 2013).

**Table 3:** Summary statistics for each measured variable, as obtained for word meanings assigned to each class

Variable		Class of word meanings								
		ANG	DIS	FEA	SAD	ANT	HAP	SUR	TRU	NEU
Valence	*M*	−0.73	−0.56	−0.33	−0.64	0.74	1.30	0.31	0.86	0.34
	*SD*	0.40	0.54	0.45	0.43	0.29	0.37	0.29	0.33	0.19
Arousal	*M*	1.49	1.29	1.43	1.46	1.29	1.65	1.12	1.27	0.88
	*SD*	0.30	0.29	0.33	0.34	0.27	0.38	0.24	0.28	0.13
Anger	*M*	1.49	0.93	0.77	0.95	0.33	0.27	0.46	0.32	0.38
	*SD*	0.44	0.40	0.28	0.32	0.12	0.10	0.15	0.10	0.10
Disgust	*M*	0.94	1.35	0.68	0.65	0.29	0.27	0.43	0.30	0.36
	*SD*	0.35	0.56	0.25	0.22	0.10	0.09	0.14	0.10	0.09
Fear	*M*	0.79	0.76	1.40	0.96	0.44	0.29	0.51	0.39	0.41
	*SD*	0.24	0.28	0.44	0.35	0.18	0.10	0.16	0.14	0.11
Sadness	*M*	1.04	0.84	0.81	1.65	0.33	0.27	0.45	0.33	0.38
	*SD*	0.32	0.35	0.31	0.49	0.12	0.09	0.14	0.11	0.10
Anticipation	*M*	0.60	0.54	0.71	0.61	1.44	1.23	0.82	1.11	0.69
	*SD*	0.17	0.16	0.22	0.17	0.31	0.31	0.19	0.28	0.11
Happiness	*M*	0.37	0.43	0.43	0.37	0.97	1.84	0.67	1.00	0.60
	*SD*	0.13	0.17	0.16	0.14	0.29	0.45	0.22	0.33	0.13
Surprise	*M*	0.85	0.77	0.84	0.80	0.78	0.80	1.07	0.64	0.61
	*SD*	0.18	0.19	0.20	0.18	0.19	0.21	0.25	0.13	0.10
Trust	*M*	0.36	0.41	0.44	0.43	0.81	1.02	0.55	1.30	0.56
	*SD*	0.11	0.14	0.15	0.14	0.21	0.31	0.14	0.37	0.10

*ANG* anger, *DIS* disgust, *FEA* fear, *SAD* sadness, *ANT* anticipation, *HAP* happiness, *SUR* surprise, *TRU* trust, *NEU* neutral, *M* mean, *SD* standard deviation

Furthermore, word meanings representing the ANG, DIS, FEA, and SAD classes were rated as relatively negative, whereas word meanings representing the ANT, HAP, and TRU classes were rated as relatively positive in terms of valence. Word meanings representing the NEU class were rated both as neutral in terms of valence and as low in terms of arousal. Word meanings representing the SUR class were also rated as predominantly neutral in terms of valence, but received various arousal ratings (Table 3). This suggests that at least some of the SUR word meanings might seem positive to some individuals, but negative to others. Indeed, word meanings assigned to the SUR class are characterized by higher variance of both valence and arousal than those assigned to the NEU class (Supplementary Figures 1 and 2). This seems to suggest that surprise is rather difficult to measure by means of self-report. In fact, earlier work on this topic emphasized that whereas most other emotions are associated with either negative or positive valence, for surprise, the case is not so clear (e.g. Noordewier & Breugelmans, 2013; Salinas et al., 2015). This has sometimes been explained by viewing surprise not as an emotion, but rather as a pre-emotional cognitive state. Specifically, Noordewier and colleagues proposed that surprise can be conceptualized as the initial response to an unexpected event, which should be differentiated from subsequent states that occur after the subject had time to evaluate the outcome of the event (Noordewier et al., 2016). Importantly, such conceptualization of surprise does not refer to valence of the outcome of the unexpected event. The outcome in itself can be positive, negative, or without a clear valence (Noordewier et al., 2016; Noordewier & Breugelmans, 2013).

It should be pointed out that different emotion classes overlap to some extent in terms of valence and arousal. For instance, ANG and SAD classes occupy the same area in the valence-arousal space. On the one hand, this means that valence and arousal alone do not determine which of these two emotions a given word represents. On the other hand, it also means that it is possible to select word meanings that represent either of these two emotions, and yet are matched in valence and arousal.

### *Supervised* and *unsupervised* study comparison

The 6000 word meanings comprising the present dataset were rated by 50.52 people on average (*min* = 38, *max* = 61) in the *unsupervised* study. Additionally, 634 of those word meanings were rated by another 24.69 people on average (*min* = 20, *max* = 28) in the *supervised* study.

**Fig. 2:**
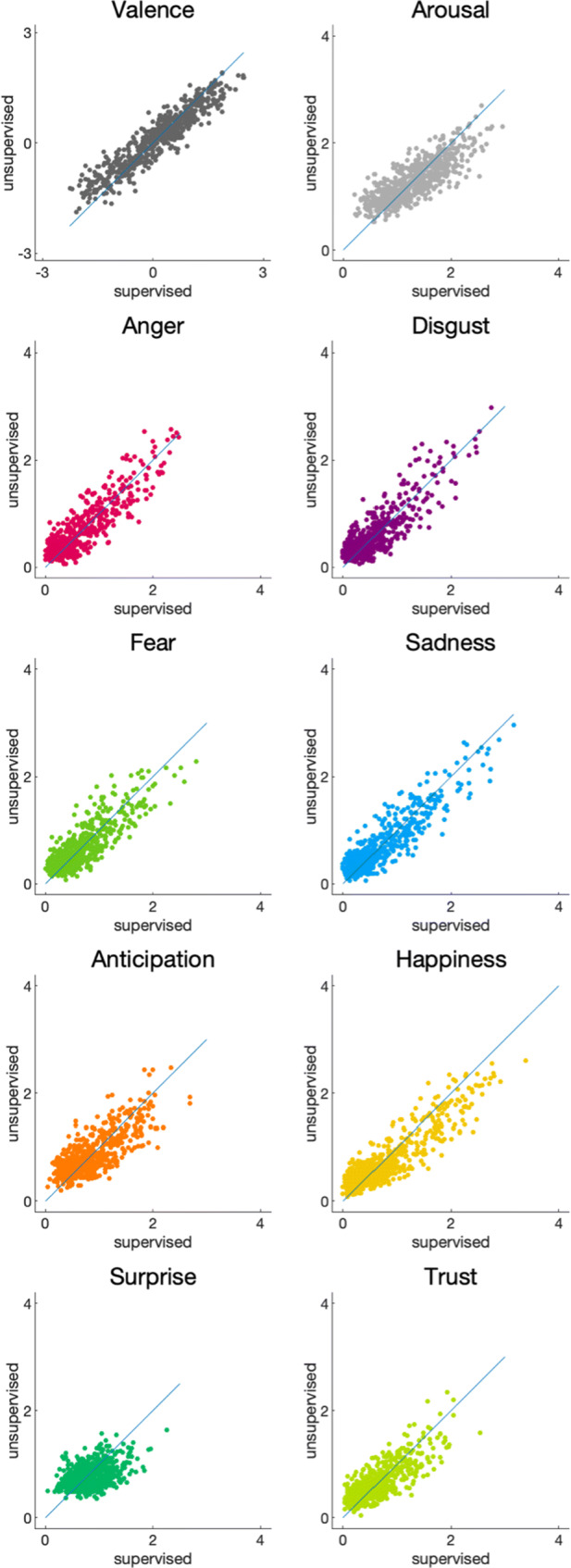
Comparison of mean ratings obtained for word meanings in the *unsupervised* and *supervised* studies. Only word meanings included in both studies (*n* = 634) are shown

A comparison of mean emotion ratings for the 634 word meanings included in both studies is presented in Fig. 2. The mean ratings obtained in both studies turned out to be similar. Pearson’s correlation coefficients calculated between the *supervised* study and the *unsupervised* study were significant for each variable of interest (valence: *r* = 0.93, *p* < 0.001; arousal: *r* = 0.81, *p* < 0.001; anger: *r* = 0.88, *p* < 0.001; disgust: *r* = 0.86, *p* < 0.001; fear: *r* = 0.84, *p* < 0.001; sadness: *r* = 0.91, *p* < 0.001; anticipation: *r* = 0.77, *p* < 0.001; happiness: *r* = 0.90, *p* < 0.001, surprise: *r* = 0.45, *p* < 0.001; and trust: *r* = 0.80, *p* < 0.001).

To further compare the ratings obtained in both studies, we performed a two-way ANOVA with *study* (two levels: supervised, unsupervised) and *rating scale* (10 levels: valence, arousal, anger, disgust, fear, sadness, anticipation, happiness, surprise, and trust) as factors. We observed no effect of *study*, *F*(1, 12660) = 1.16, *p* = 0.281, *η*^*2*^ < 0.001, nor an interaction effect between *study* and the *rating scale*, *F*(9, 12_,_660) = 1.30, *p* = 0.233, *η*^*2*^ < 0.001. This confirms that the ratings obtained in the two studies were overall comparable.

### Demographic subgroups: gender and age as example use cases

The present dataset contains detailed information on participants’ gender, age, place of residence, education, relationship status, employment status, income, as well as political views. To demonstrate how this information can be used, we split the ratings obtained from all the participants according to their gender and age. Only the *unsupervised* ratings were used, as they were available for all the 6000 word meanings.

In the first example we split the ratings based on participants’ gender (*female*–*male*). Overall, the word meanings were rated by 31.30 females on average (*min* = 16, *max* = 49, *median* = 31, *mode* = 32) and by 19.23 males on average (*min* = 8, *max* = 33, *median* = 19, *mode* = 19).

In the second example, we split the ratings based on participants’ age (*young*–*old*). The participants were divided so as to form two groups of roughly the same size. Hence, all participants younger than 35 years at the time of the study were considered *young*, whereas the remaining participants were considered *old*. Overall, the word meanings were rated by 21.48 younger individuals on average (*min* = 9, *max* = 35, *median* = 21, *mode* = 21) and by 29.04 older individuals on average (*min* = 14, *max* = 44, *median* = 29, *mode* = 30).

For most of the word meanings, the mean ratings obtained from the demographic groups described above were similar. A comparison of mean emotion ratings obtained from the female and male groups is presented in Supplementary Figure 4. Pearson’s correlation coefficients calculated between the ratings given by *female* and *male* individuals were significant for each variable of interest (0.31 < *r* < 0.88, *p* < 0.001 for all compared variables). The same holds for a comparison of mean ratings obtained from the *young* and *old* groups, depicted in Supplementary Figure 5. Pearson’s correlation coefficients calculated between the ratings given by younger and older individuals were significant for each variable of interest (0.31 < *r* < 0.90, *p* < 0.001 for all compared variables).

One possible use of the demographic information provided with the present dataset is to select word meanings based on the ratings provided by a specific demographic group. For instance, Tables 4 and 5 list word meanings rated highest in terms of each measured variable by each of the compared groups. Furthermore, the demographic information can also be used to select word meanings rated most dissimilarly by the two groups (see Supplementary Figures 4 and 5). While most researchers will find the ratings obtained from the whole group of participants sufficient for their needs, making use of the demographic information can prove useful in more specialized applications.

**Table 4:** Word meanings with the highest ratings in females and males, respectively. Corresponding English word meanings are provided in parentheses. English word meanings were derived from the Princeton Wordnet, based on the cross-lingual relation between Polish and English synsets

	Top word meanings			
	Females		Males	
Variable	Word meaning	Rating	Word meaning	Rating
Anger	arogancki (arrogant; chesty; self-important)	3.04	hałaśliwy (loud)	2.95
	powolność (sluggishness)	2.91	niekoleżeński (inimical; unfriendly)	2.85
	zrzędliwość (querulousness)	2.86	spalony (burned; burnt)	2.80
Disgust	oślizgły (bad; spoiled; spoilt; gluey; glutinous; gummy; mucilaginous; pasty; sticky; viscid; viscous; slithery)	3.25	wstrętny (cursed; curst)	3.26
	obleśnie (lewdly; obscenely)	3.08	śmierdzący (fetid; foetid; foul; foul-smelling; funky; ill-scented; noisome; smelly; stinking)	2.94
	zgniły (rotten)	3.04	zgniły (rotten)	2.92
Fear	zagrożenie (danger)	3.04	narażenie (exposure)	2.75
	przepaść (chasm)	2.94	rozjuszony (angered; enraged; furious; infuriated; maddened)	2.71
	przestraszny (fearful; frightful)	2.90	niebezpieczny (dangerous; unsafe)	2.60
Sadness	żałobny (doleful; mournful)	3.37	zmarły (dead person; dead soul; deceased; deceased person; decedent; departed)	3.04
	pogrzeb (end; last)	3.17	nieszczęśliwie (unhappily)	2.89
	kondolencyjny (communicative)	3.17	zapłakany (tearful)	2.88
Anticipation	los (draw; lot; ticket)	2.77	konsultacja (public discussion; ventilation)	2.73
	start (scratch; scratch line; start; starting line)	2.77	wstęp (introduction)	2.53
	zwiastun (preview; prevue; trailer)	2.76	zdrowo (good; well)	2.52
Happiness	słońce (good weather)	3.44	śliczny (beautiful)	3.20
	przesympatyczny (nice)	3.34	zadowolony (content; contented; glad)	3.06
	czekolada (milk chocolate)	3.25	przeszczęśliwy (happy)	2.95
Surprise	wizyta (visit)	2.96	niespodziewany (unexpected)	2.24
	nadprogramowy (additional; extra)	2.58	wizyta (visit)	2.18
	niespodziewany (unexpected)	2.43	nieprzewidziany (ad-lib; spontaneous; unwritten)	2.14
Trust	stabilny (stable)	2.84	partnerski (cooperative)	2.56
	lojalność (loyalty; trueness)	2.70	senior (patriarch)	2.47
	dyskrecja (concealment; privacy; privateness; secrecy)	2.67	mądrość (wisdom)	2.44

**Table 5:** Word meanings with the highest ratings in younger and older individuals, respectively. Corresponding English word meanings are provided in parentheses. English word meanings were derived from the Princeton Wordnet, based on the cross-lingual relation between Polish and English synsets

	Top word meanings			
	Younger individuals		Older individuals	
Variable	Word meaning	Rating	Word meaning	Rating
Anger	złość (anger; choler; ire;distemper; ill humor; ill humour)	3.05	hałaśliwy (loud)	2.88
	powolność (sluggishness)	3.00	partacki (botched; bungled)	2.79
	gówniarsko (-)	2.89	kretyński (stupid; idiotic; imbecile; imbecilic)	2.72
Disgust	grzybica (tinea unguium)	3.21	oślizgły (bad; spoiled; spoilt; gluey; glutinous; gummy; mucilaginous; pasty; sticky; viscid; viscous; slithery)	3.25
	nieświeży (stale)	3.16	śmierdzący (fetid; foetid; foul; foul-smelling; funky; ill-scented; noisome; smelly; stinking)	3.12
	fekalny (faecal; fecal)	3.13	syfiasto (badly; ill; poorly)	2.97
Fear	narażenie (exposure)	3.50	kleszczowy (artefactual; artifactual)	3.11
	przeklęty (cursed; curst)	2.80	rozjuszony (angered; enraged; furious; infuriated; maddened)	2.86
	syk (fizzle; hiss; hissing; hushing; sibilation)	2.72	przepaść (chasm)	2.85
Sadness	pogrzeb (end; last)	3.41	zmarły (dead person; dead soul; deceased; deceased person; decedent; departed)	3.17
	przedpogrzebowy (funerary; special; antecedent)	3.27	ceremonia (attending; attention)	3.14
	żałoba (bereavement; mourning)	3.19	żałobniczka (griever; lamenter; mourner; sorrower)	3.12
Anticipation	odpowiedź (counsel; counseling; counselling; direction; guidance)	2.68	zadowolony (content; contented; glad)	2.74
	zwiastun (preview; prevue; trailer)	2.65	losowy (random)	2.61
	przysmak (dainty; delicacy; goody; kickshaw; treat)	2.58	los (draw; lot; ticket)	2.59
Happiness	uszczęśliwiony (happy)	3.20	gromki (sudden; loud)	3.26
	uśmiechnięty (beamish; smiling; twinkly)	3.14	zadowolony (content; contented; glad)	3.17
	piknik (field day; outing; picnic)	3.10	słońce (good weather)	3.12
Surprise	wizyta (visit)	2.81	niespodziewany (unexpected)	2.82
	zaskoczenie (surprise)	2.20	wizyta (visit)	2.59
	cudaczny (bizarre; eccentric; flakey; flaky; freakish; freaky; gonzo; off-the-wall; outlandish; outre; weird)	2.19	nadprogramowy (additional; extra)	2.34
Trust	partnerski (cooperative)	2.73	stabilny (stable)	3.00
	babcia (old woman)	2.58	lojalność (loyalty; trueness)	2.89
	przytulanka (-)	2.52	pasy (pedestrian crossing; zebra crossing)	2.58

## Discussion

Emotion lexicons or lists of words are widely used and can benefit various disciplines of research. On the one hand, they can be useful in research concerned with the psychology of emotion and its impact on other cognitive processes (Barrett et al., 2007; Lindquist, 2017). On the other hand, they can be valuable to research focused on automatic detection of emotion in natural language, where they can inform computational models that process large amounts of text (Cowen & Keltner, 2021; Dodds et al., 2015; Reagan et al., 2017).

However, the availability of high-quality lexicons remains limited, as the data collection process is typically very effortful and expensive. In the present work we have outlined the general approaches to create such lexicons, rooted in traditions of different scientific disciplines. In our approach we combine the insight contributed by those various disciplines of research to introduce the Emotion Meanings dataset—a novel, versatile dataset of 6000 Polish word meanings annotated in terms of emotion.

### The strengths of emotion lexicons originating from the psychological tradition

Perhaps the most characteristic feature of emotion lexicons designed for use in psychology and cognitive science is that such lexicons are based on data collected from a large group of people, rather than a single individual or at most a few “experts.” This approach is grounded in the view—common in psychology—that emotion is a complex phenomenon, experienced subjectively, observable and measurable only indirectly (Mauss & Robinson, 2009; Moors, 2009). Thus, almost any word might elicit different emotional responses from different people, especially that due to personal experience a seemingly commonplace word may evoke a strong emotional reaction in an individual (Brosch et al., 2010). By adopting this approach in the present work, we have been able to determine whether and to what extent participants differ in their emotional response. For each word meaning, we provide both the central tendency and dispersion measures, as well as individual ratings given by each participant. Our results show the ratings to be highly robust, demonstrating the high quality of the collected data. In particular, the ratings obtained in the *supervised* and *unsupervised* groups were highly similar.

Furthermore, another benefit of collecting many ratings for each word is that such data can be used to investigate various demographically specific subgroups. As language evolves, the way people use some words, together with their emotional impact, continues to shift (Xu et al., 2017). Similarly, the way we use language depends on our personal experiences and on what demographic or social group we belong to. Previous research provided substantial evidence for gender differences in emotional response and perception (Stevens & Hamann, 2012). Similarly, age was shown to impact the way we process emotional information (Grühn & Scheibe, 2008; Grühn & Smith, 2008; Keil & Freund, 2009; Mather & Carstensen, 2005). Our dataset provides the means to capture these subtle differences by the inclusion of detailed demographic information on each participant. Although we found the annotations to be relatively stable for females and males, as well as younger and older participants, we share both summary data and individual ratings contributed by each participant. This gives the researchers freedom to use the present dataset in various ways and explore it from a different angle, for example, to investigate data from demographically specific subgroups.

It should be noted that in the present work we have not explored the impact of the remaining demographic variables on the emotional assessment of words and their meaning. With this dataset we share the following information on each participant: place of residence, education, relationship status, employment status, income, as well as political views. We hope these data will enable further research and motivate other, more refined analyses of various demographically specific subgroups. Thus, the present dataset can help address research questions about the population as a whole, and about specific demographic or social groups. This can be useful not only in psychology, but also in various natural language processing applications.

### The strengths of emotion lexicons originating from the natural language processing tradition

The greatest strength of emotion lexicons designed for natural language processing applications is that such lexicons typically comprise word meanings or word senses (Fellbaum, 1998, 2006) rather than words. Indeed, it is a particular meaning of a word (rather than the word itself) that conveys emotion. A word placed out of context can be interpreted in various ways, depending on what we mean by it (De Deyne et al., 2019). In turn, different interpretations of the same word may bring different associations to mind and give rise to different emotions. For instance, in response to a Polish word *drogi*, some people might take it to mean *dear*, while others might consider another of its meanings, namely, *expensive*. In such a case, pooling the annotations together and calculating the mean would most likely bring us to the (false) conclusion that the word is emotionally neutral. Our approach to use word meanings as elementary units of the dataset allows us to avoid this pitfall.

Having access to large linguistic resources is certainly powerful and has many desirable implications. First, the present dataset is directly linked to plWordNet (Piasecki et al., 2009), a large lexico-semantic network that interlinks words by means of lexical and conceptual relations. Furthermore, word meanings are accompanied by relevant linguistic data, derived from other open-source resources. Thus, researchers interested in word meanings of particular properties are not restricted to rely on our dataset only, but can browse the vast amount of data included in plWordNet and other resources. In particular, it has been demonstrated that similar words (e.g. words that frequently occur together) are likely to have similar emotional connotations (Van Rensbergen et al., 2016). Thus, researchers may infer the emotional properties of a word not included in the present dataset, based on relations between this word and others, for which we provide complete data. However, such automatically derived emotion annotations should be validated against human data (Brysbaert et al., 2017; Van Rensbergen et al., 2016).

Similarly, thanks to a direct mapping between the plWordNet (Piasecki et al., 2009) and the Princeton WordNet (Fellbaum, 1998; Miller, 1995), the present dataset could be useful in research involving multiple languages. The Princeton WordNet was conceived in 1986 at Princeton University and is the first and most widely known such resource in the world. However, similar resources are being developed for other languages. The Global WordNet Association curates a list of all available wordnets.^11^ Thus, it should be possible to use our dataset together with information derived from a variety of other languages.

## Conclusions and limitations

In summary, several properties of the Emotion Meanings dataset make it a rich and valuable resource, likely to facilitate research across several fields of scientific study. First, we provide information on the emotional properties of each word meaning in line with the two most widely acknowledged theoretical frameworks: *dimensional*(valence and arousal; Bradley & Lang, 1994; Osgood et al., 1957; Russell & Mehrabian, 1977) and *categorical*(anger, disgust, fear, sadness, anticipation, happiness, surprise, and trust; Ekman, 1992; Ortony & Turner, 1990; Plutchik, 1982). Importantly though, while we combine the insights contributed by various disciplines, the applicability of the Emotion Meanings dataset to some disciplines (e.g. computational linguistics, natural language processing) is limited due to its rather small size. Future studies could focus on building high-quality, large-scale lexicons that could be used across scientific disciplines. Second, the dataset is directly linked to the Polish wordnet (plWordNet)and—by extension—to the Princeton WordNet. Thus, it can contribute to the advancement of multilingual research. However, it should be pointed out that there is no simple one-to-one mapping between different natural languages. In fact, in our case, the mapping was based on the cross-lingual relation between Polish and English synsets (i.e. sets of word meanings representing the same concept). Future studies could either develop tools for more precise mapping between word meanings across these two languages, or—at the very least—take this limitation into consideration in the study design process. Finally, we share both summary data and individual data, together with detailed demographic information on each individual participant. These data can be used in many potential ways, depending on the particular case. Yet, it should be noted that the amount of data collected in the present project allows for very rough comparisons only (e.g. females vs. males, younger vs. older individuals). Future studies may be interested in more fine-grained comparisons that would certainly require more data. Altogether, this dataset provides a versatile resource that can be used for emotion research in psychology, cognitive science, psycholinguistics, computational linguistics, and natural language processing. To the best of our knowledge, it is the first such project conducted in Poland and quite certainly one of the few conducted worldwide.

## Supplementary Information

ESM 1 (DOCX 2838 kb)

## Footnotes

1 http://plwordnet.pwr.wroc.pl/wordnet

2 https://wordnet.princeton.edu

3 http://plwordnet.pwr.wroc.pl/wordnet/stats

4 http://nlp.pwr.wroc.pl/plwordnet/license/

5 https://ling.pl

6 https://plwordnet.pwr.wroc.pl/wordnet/synset/3807

7 https://plwordnet.pwr.wroc.pl/wordnet/synset/256795

8 https://plwordnet.pwr.wroc.pl/wordnet/synset/291598

9 https://plwordnet.pwr.wroc.pl/wordnet/synset/359869

10 https://stat.gov.pl/en

11 http://globalwordnet.org/resources/wordnets-in-the-world
