# Supplementary material for: Emotion norms for 6000 Polish word meanings with a direct mapping to the Polish wordnet
Source: Behav Res Methods. 2021 Dec 10;54(5):2146–61. doi: 10.3758/s13428-021-01697-0 (PMC9579083; doi:10.3758/s13428-021-01697-0)

# Supplementary Figures

**Supplementary Figure 1.** Distribution of the valence ratings (means and standard deviations) for word meanings assigned to each class. In each case, the darker color represents word meanings belonging to a given class, whereas the light gray represents the remaining word meanings. Abbreviations: ANG - anger, DIS - disgust, FEA - fear, SAD - sadness, ANT - anticipation, HAP - happiness, SUR - surprise, TRU - trust, NEU - neutral, *M* - mean, *SD* - standard deviation.


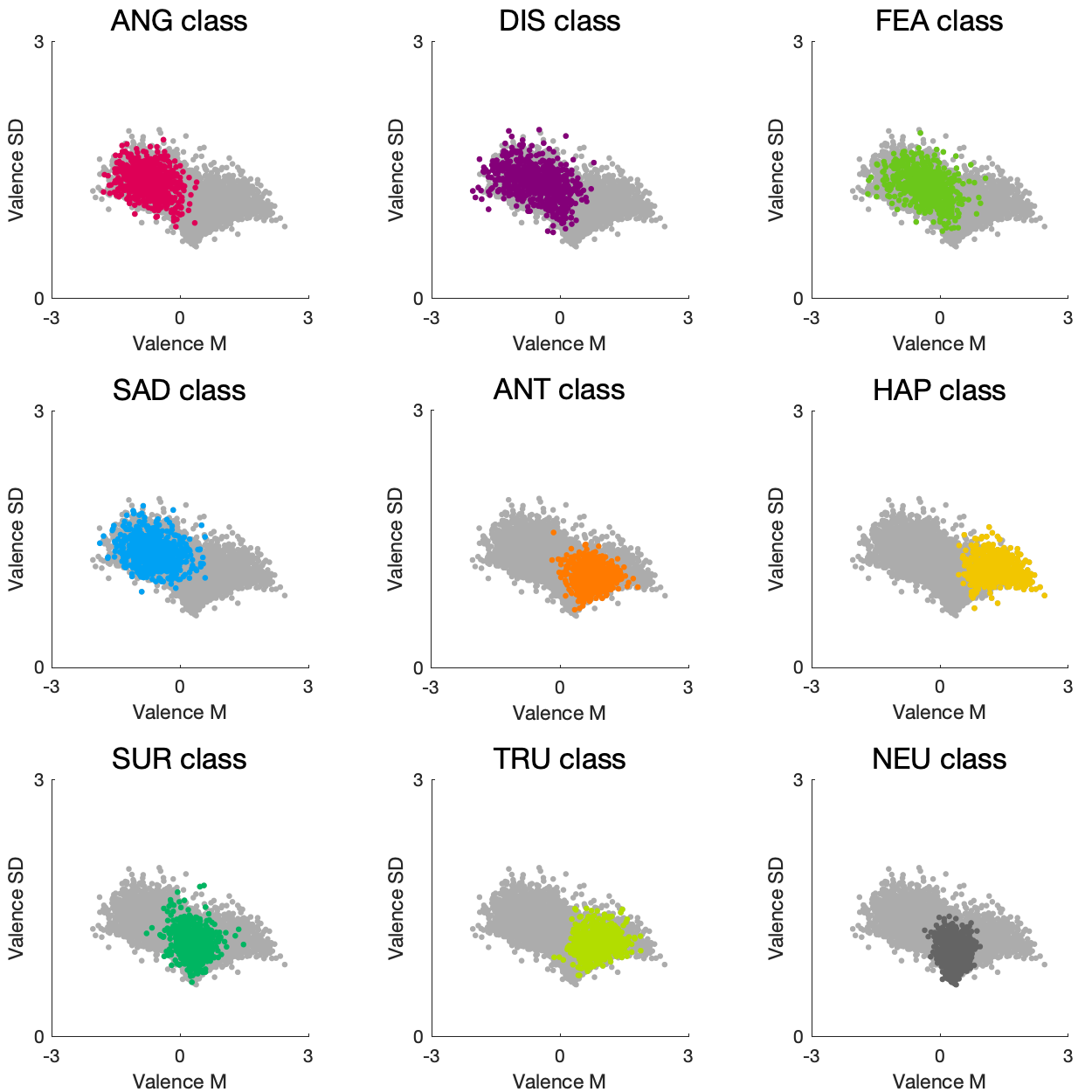


**Supplementary Figure 2.** Distribution of the arousal ratings (means and standard deviations) for word meanings assigned to each class. In each case, the darker color represents word meanings belonging to a given class, whereas the light gray represents the remaining word meanings. Abbreviations: ANG - anger, DIS - disgust, FEA - fear, SAD - sadness, ANT - anticipation, HAP - happiness, SUR - surprise, TRU - trust, NEU - neutral, *M* - mean, *SD* - standard deviation.


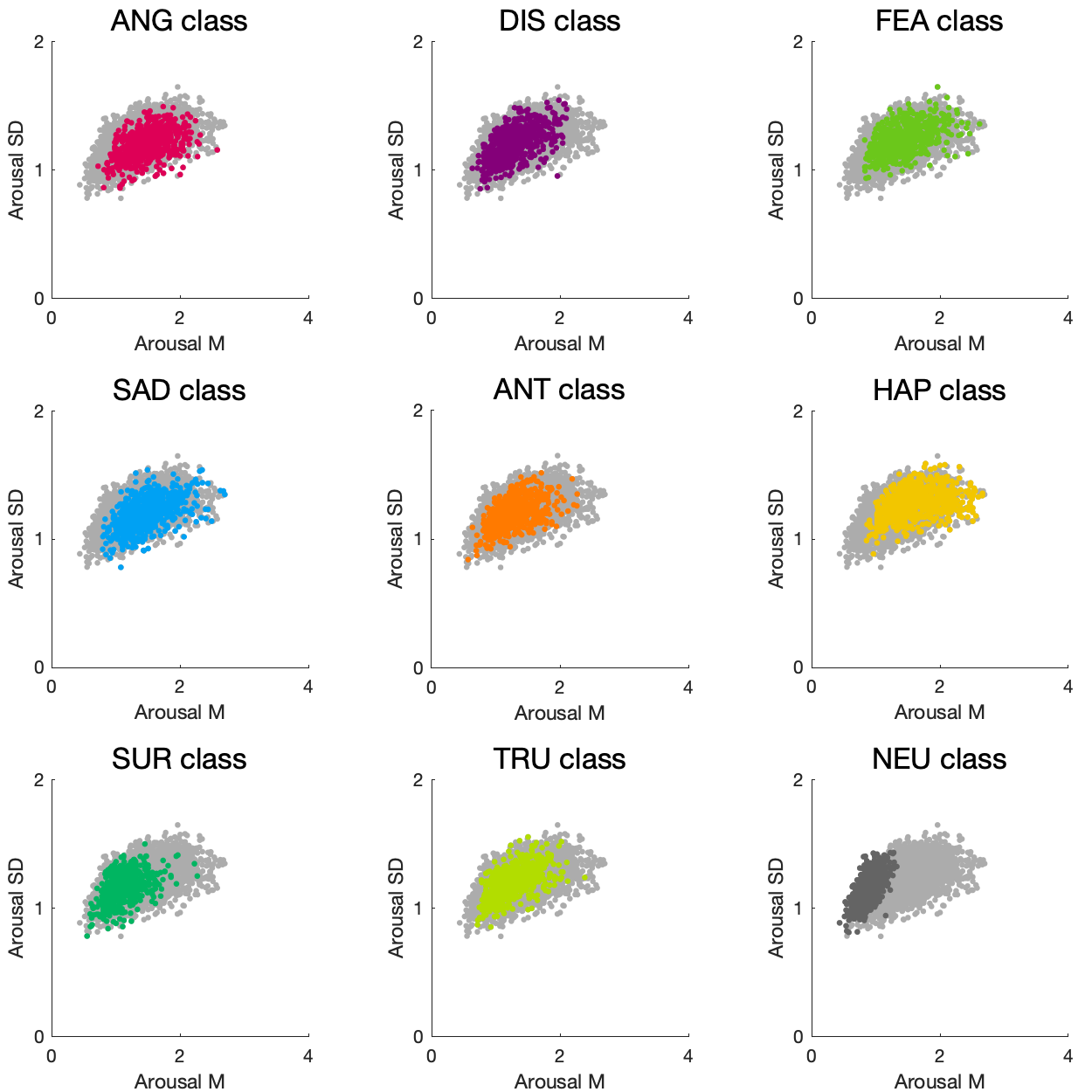


**Supplementary Figure 3.** The distribution of mean valence, arousal, anger, disgust, fear, sadness, anticipation, happiness, surprise, and trust ratings for word meanings assigned to each class. Abbreviations: ANG - anger, DIS - disgust, FEA - fear, SAD - sadness, ANT - anticipation, HAP - happiness, SUR - surprise, TRU - trust, NEU - neutral.

**
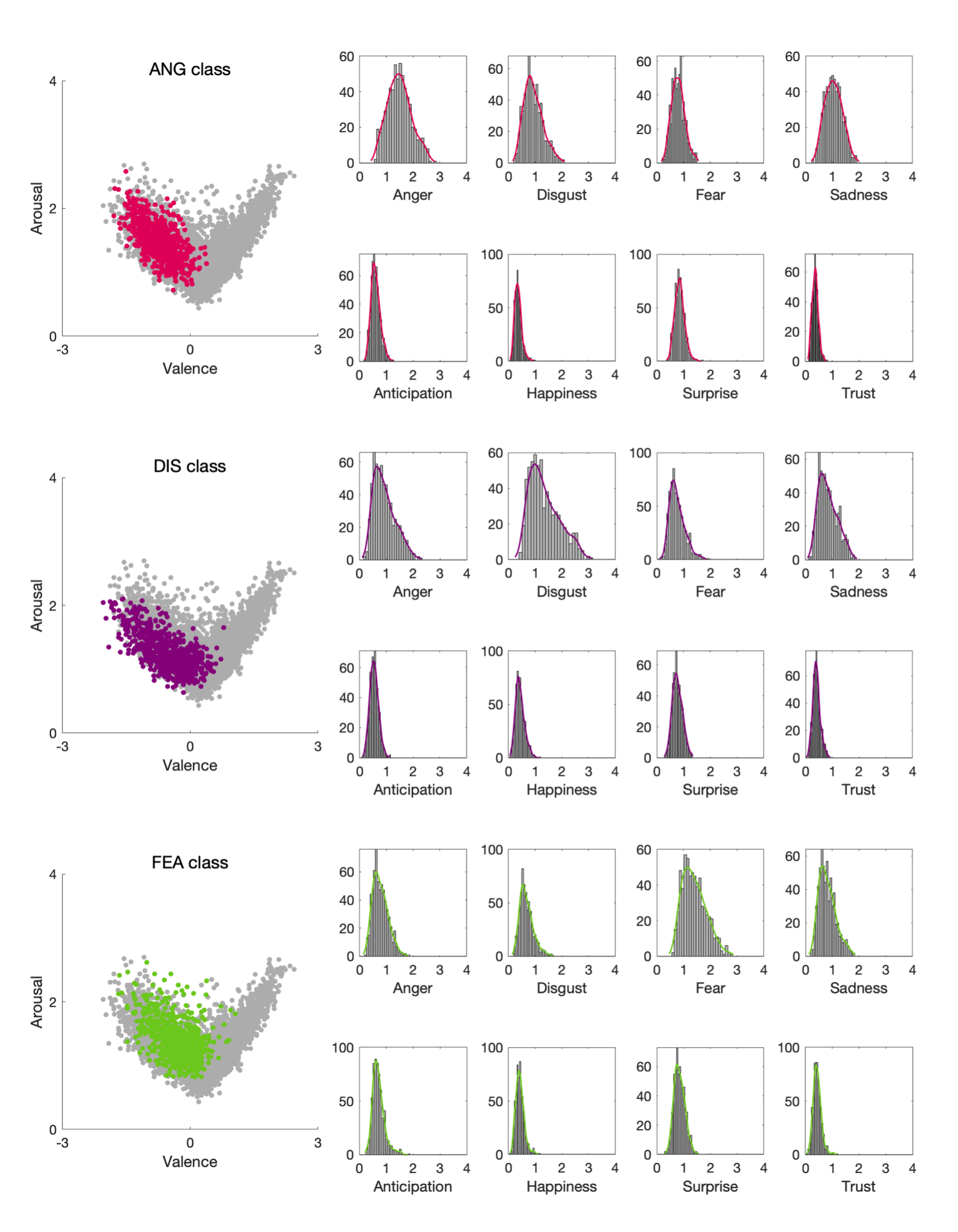
**

**
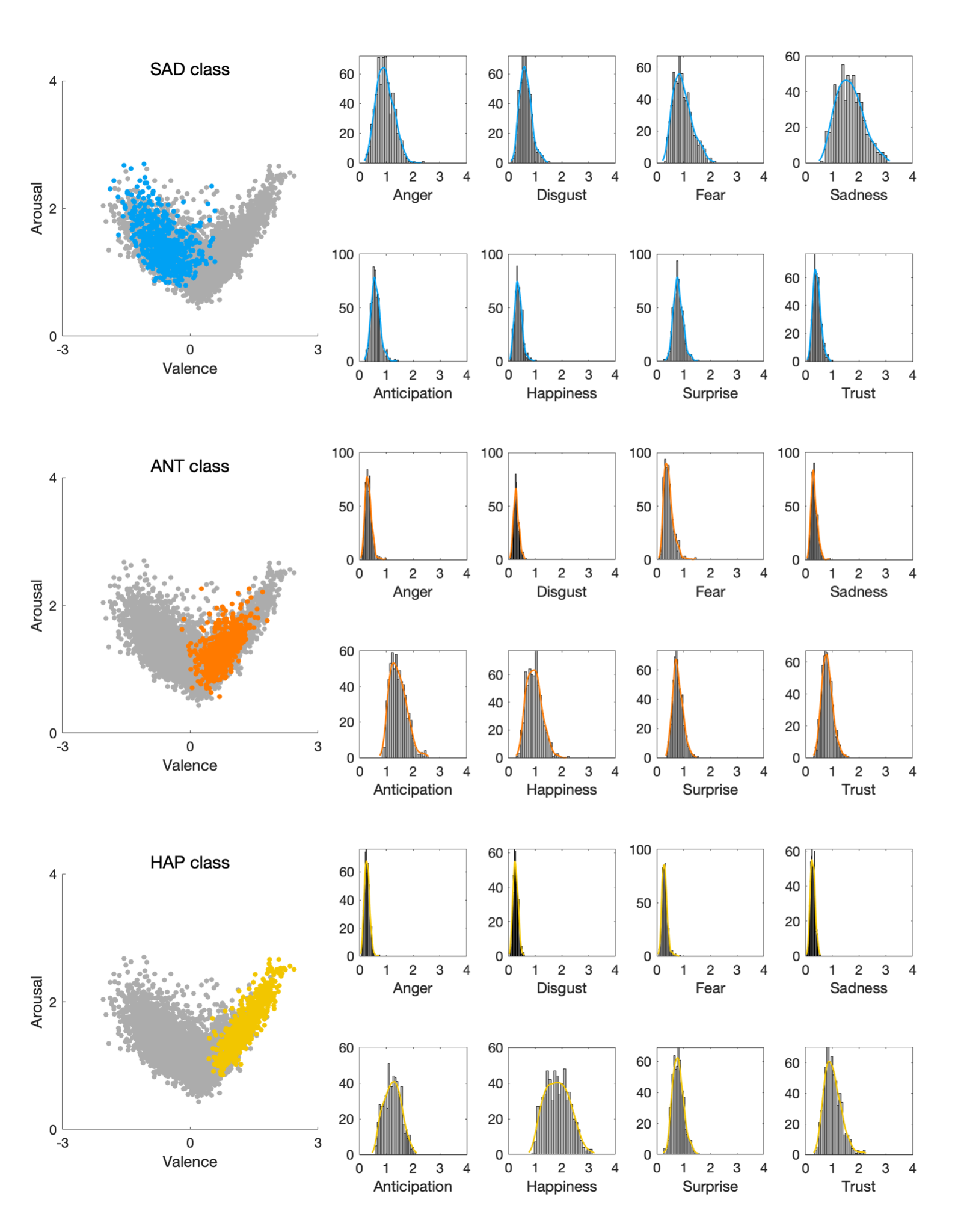
**

**
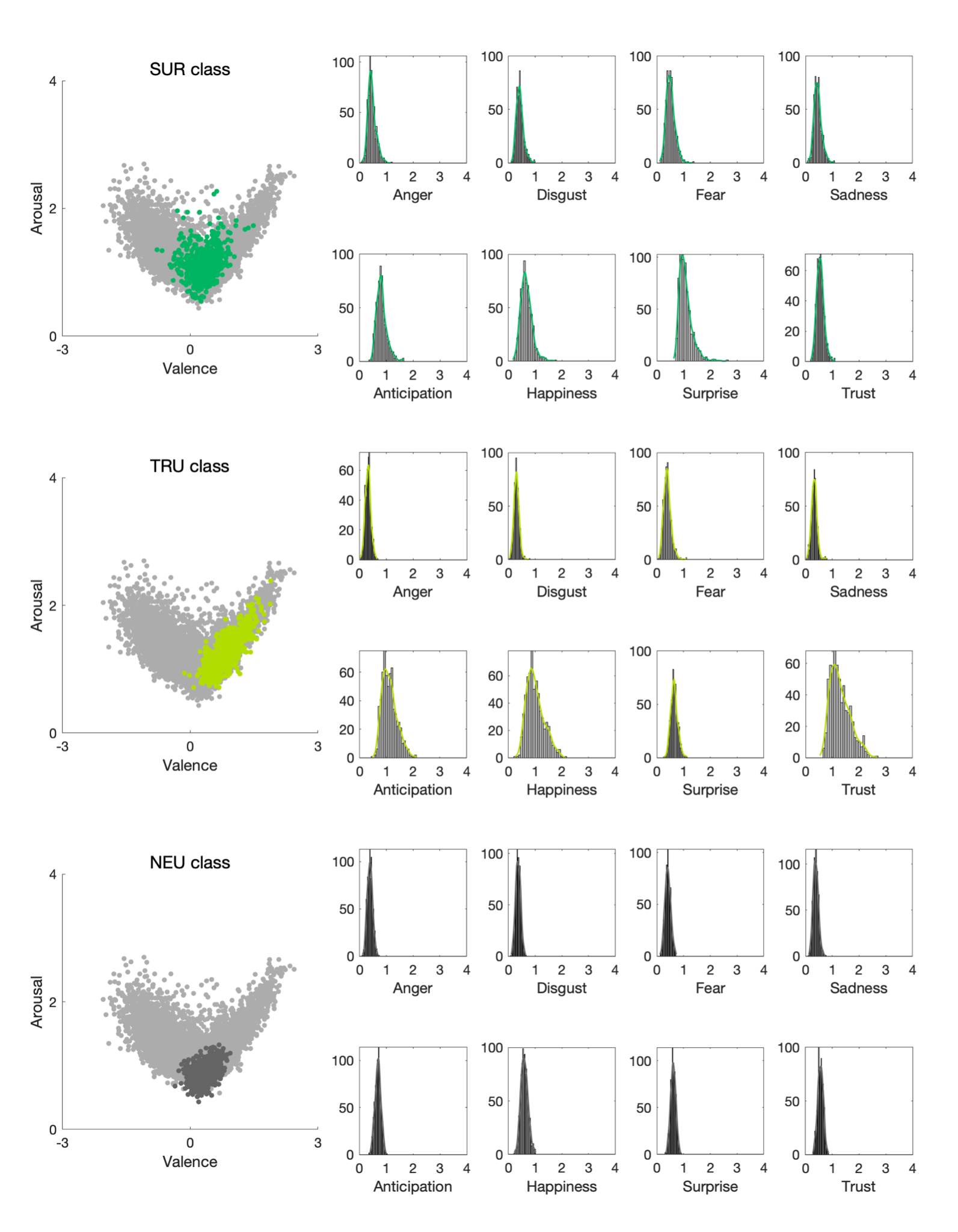
**

**Supplementary Figure 4.** Comparison of mean ratings given by females and males. The darker color represents the top 100 word meanings rated most dissimilarly by the two groups, whereas the light beige represents the remaining word meanings.

**
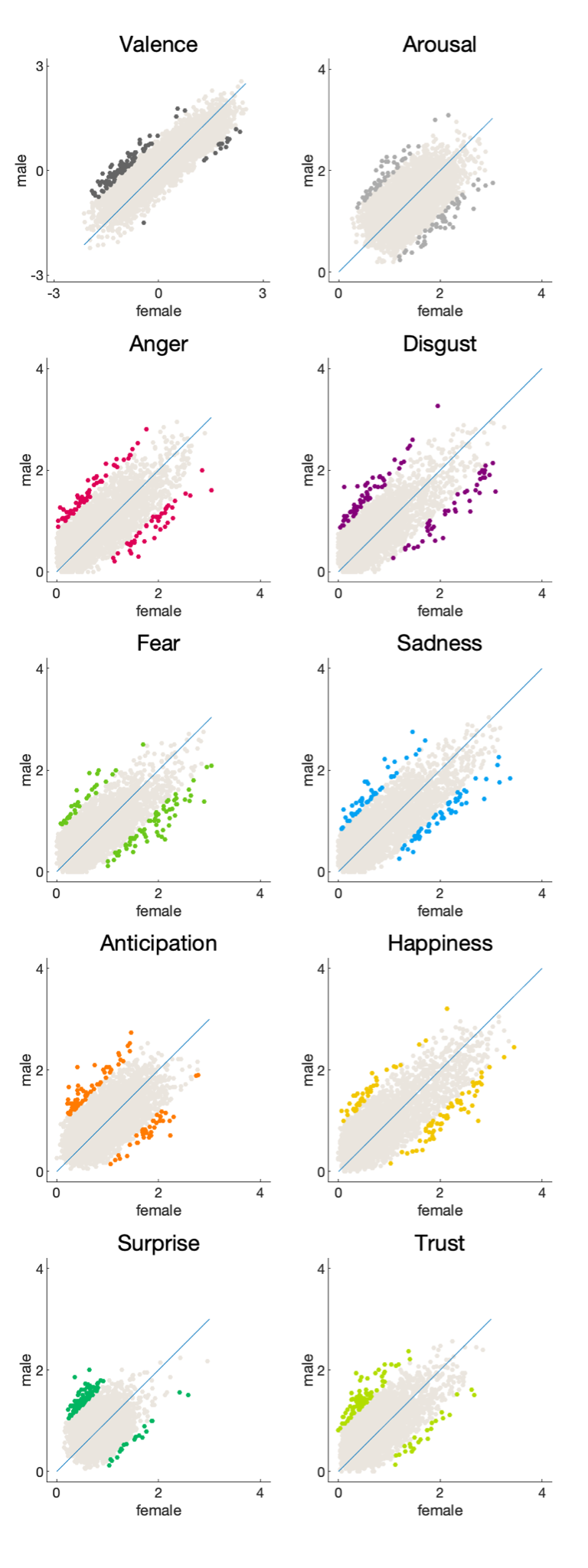
**

**Supplementary Figure 5.** Comparison of mean ratings given by younger and older individuals. The darker color represents the top 100 word meanings rated most dissimilarly by the two groups, whereas the light beige represents the remaining word meanings.


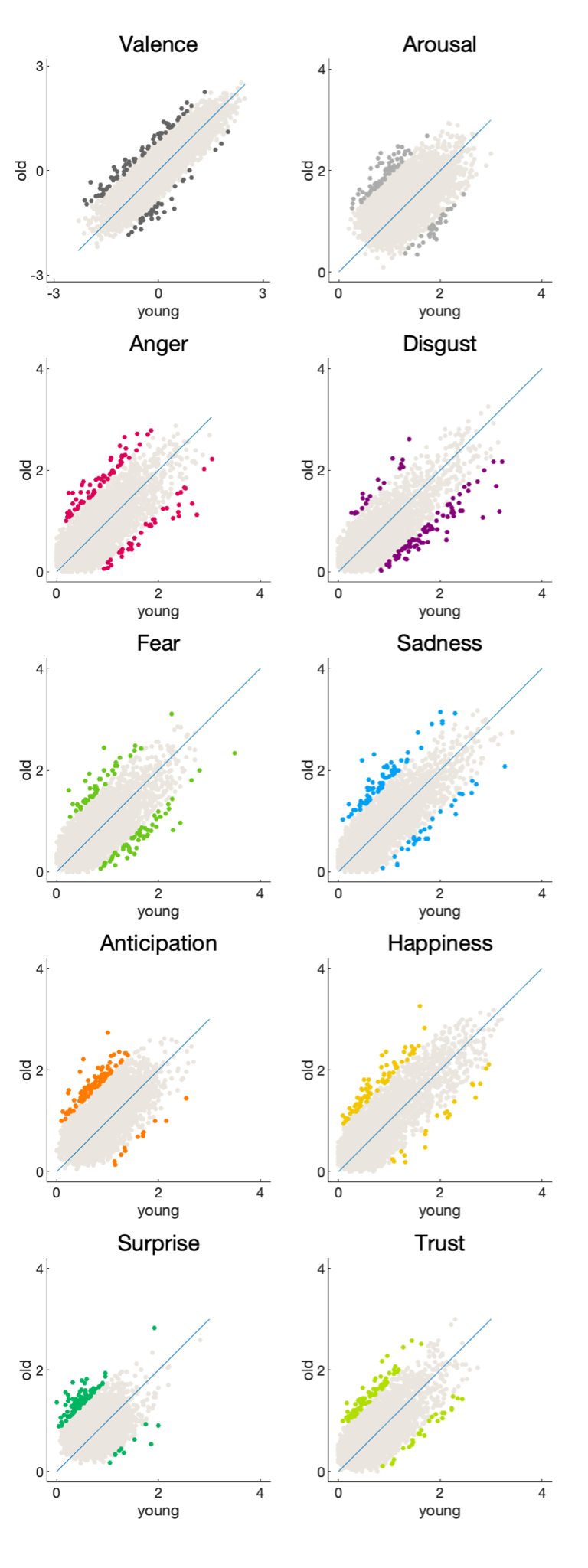

Supplement: Supplementary file 1 — (DOCX 2838 kb) [file 13428_2021_1697_MOESM1_ESM.docx]
